# Glucocorticoid receptor hypersensitivity enhances inflammatory signaling and inhibits cell cycle progression in porcine PBMCs

**DOI:** 10.3389/fimmu.2022.976454

**Published:** 2022-11-24

**Authors:** Zhiwei Li, Frieder Hadlich, Klaus Wimmers, Eduard Murani

**Affiliations:** ^1^ Institute of Genome Biology, Research Institute for Farm Animal Biology (FBN), Dummerstorf, Germany; ^2^ Faculty of Agricultural and Environmental Sciences, University Rostock, Rostock, Germany

**Keywords:** glucocorticoid receptor hypersensitivity, transcriptome, porcine PBMCs, lipopolysaccharide, inflammatory signaling, cell cycle progression, pattern recognition receptors

## Abstract

The consequences of glucocorticoid receptor (GR) hypersensitivity during infection have so far received little attention. We previously discovered that a natural gain-of-function Ala610Val substitution in the porcine GR aggravates response of pigs to lipopolysaccharide (LPS)-induced endotoxemia, which can be alleviated by dexamethasone (DEX) pretreatment. In this work, we investigated the relevant molecular basis of these phenotypes by transcriptomic profiling of porcine peripheral blood mononuclear cells (PBMCs) carrying different GR genotypes, in unstimulated conditions or in response to DEX and/or LPS *in vitro*. The Val allele differentially regulated abunda+nt genes in an additive-genetic manner. A subset of more than 200 genes was consistently affected by the substitution across treatments. This was associated with upregulation of genes related i.a. to endo-lysosomal system, lipid and protein catabolism, and immune terms including platelet activation, and antigen presentation, while downregulated genes were mainly involved in cell cycle regulation. Most importantly, the set of genes constitutively upregulated by Val includes members of the TLR4/LPS signaling pathway, such as *LY96*. Consequently, when exposing PBMCs to LPS treatment, the Val variant upregulated a panel of additional genes related to TLR4 and several other pattern recognition receptors, as well as cell death and lymphocyte signaling, ultimately amplifying the inflammatory responses. In contrast, when stimulated by DEX treatment, the Val allele orchestrated several genes involved in anti-inflammatory responses during infection. This study provides novel insights into the impact of GR hypersensitivity on the fate and function of immune cells, which may be useful for endotoxemia therapy.

## Introduction

Glucocorticoids (GCs) are a class of steroid hormones produced by the hypothalamic–pituitary–adrenal (HPA) axis (1). As end products of the HPA axis, these molecules participate in a range of biological processes that help organisms cope with stress conditions and restore homeostasis (2). Proper GC action is essential for efficient control of many immune reactions, and to prevent damage from maladapted responses (3). Clinically, GCs are long-standing anti-inflammatory agents that are among the most commonly prescribed drugs in human and veterinary medicine, and are widely used for the treatment of inflammatory and autoimmune diseases (4). Accordingly, dysregulation of GC signaling is observed in several inflammatory diseases, such as sepsis - one of the leading causes of mortality worldwide, and complicates their therapy (5, 6). Endotoxin (Lipopolysaccharide – LPS) is a component of the outer membrane of gram-negative bacteria, and is the most potent pathogen-associated molecular pattern (PAMP) inducing a host of physiological responses including a strong inflammatory reaction as well as a homeostatic activation of the HPA axis (7). Endotoxemia is frequently observed in patients with sepsis (8), and experimentally-induced endotoxemia *via* LPS administration is commonly used to study the pathobiology of sepsis, even though there are some limitations (9).

GCs exert their effects mainly *via* the glucocorticoid receptor (GR), a ligand-inducible transcription factor (TF) belonging to the nuclear receptor superfamily (10). Thus, relevant mechanisms of GR signaling and their regulation are under intense investigation using a variety of approaches (11). These studies have demonstrated the necessity of retaining GR dimerization, which is important for receptor activation, and established the importance of enhanced GR expression and TF activity for adequate anti-inflammatory effects. For example, GR dimerization deficiency in GR^dim^ mice resulted in more severe inflammation and sepsis (12, 13), whereas enhancement of GR expression and transactivation activity attenuated LPS-induced endotoxic shock and lethal inflammation (14, 15). These examples emphasize the positive functional role of the GR in anti-inflammatory processes.

In contrast, our previous findings revealed that this association is not always favorable. We observed that the gain-of-function of GR caused by a natural Ala610Val substitution (GR_Ala610Val_) increased the susceptibility of pigs to LPS-induced endotoxemia (16, 17). This GR variant showed higher ligand-binding affinity and transactivation activity *in vitro* (17). However, it caused aggravated cytokine production, thrombocytopenia, sickness behaviors, anorexia, and metabolic alterations in pigs upon endotoxin challenge (16). The underlying mechanisms behind this negative influence of GR hypersensitivity remain unclear. Their exploration may shed new light on glucocorticoid receptor dysregulation in inflammatory diseases. Importantly, under baseline conditions hypersensitivity of the mutated GR manifests largely only by decreased cortisol level (18), suggesting that GR hypersensitivity may be a complication in inflammatory diseases that has remained largely unrecognized. GR_Ala610Val_ provides an exceptional model not only because it is a unique natural gain-of-function GR variant, but the pig model appears particularly relevant because, unlike rodents, pigs feature similar sensitivity to endotoxemia compared to humans (19).

This study set out to investigate the influence of the gain-of-function GR_Ala610Val_ genetic variant on the transcriptomic programs of porcine peripheral blood mononuclear cells (PBMCs), at baseline and in response to stimulation by dexamethasone (DEX) and LPS, separately and jointly. To achieve this, porcine PBMCs carrying each of three GR genotypes (Ala/Ala, Ala/Val, Val/Val) were divided into four groups and treated with either vehicle (CON), DEX, LPS, or LPS+DEX for two hours. Next, mRNA sequencing was carried out to elucidate Val allele-associated transcriptomic responses, and the relevant signaling pathways and biological events were subsequently explored using different computational tools. The results are anticipated to improve our understanding of the impact of GR hypersensitivity in the contexts of GC-mediated therapy, immune stimulation, and crosstalk of immune and GR signaling. The knowledge gained may benefit the health and welfare of both humans and farm animals.

## Materials and methods

### Sampling, treatment, and mRNA sequencing

The mRNA sequencing data presented and analyzed in the current report were first published and all the sampling and treatment procedures were done in our previous study (20). Briefly, PBMCs were obtained from 24 purebred German Landrace pigs (≈170 days old) with equal numbers of males and females and balanced for the three GR genotypes (Ala/Ala, Ala/Val, and Val/Val). The animals were reared at the FBN experimental pig farm (Dummerstorf, Germany) according to the German Law of Animal Protection. PBMCs from each animal were distributed into four treatment groups and treated for 2 h at 37°C in a 5% CO_2_ atmosphere with: vehicle (CON group); DEX (5 nM); LPS (10 μg/mL); and LPS + DEX, respectively. The mRNA sequencing was performed using the Illumina HiSeq 2500 platform at the FBN sequencing facility as described previously (20). The resultant sequencing data were first submitted to the ArrayExpress repository (https://www.ebi.ac.uk/arrayexpress) with accession number E-MTAB-9808, as outlined in our earlier study (20).

### Differential expression analysis

Prior to analysis, genes with normalized counts ≥5 in fewer than eight of the samples were removed. Differential expression analysis was then performed using the R package DESeq2 version 1.32.0 (21). Principal component analysis (PCA) identified four outlier animals, which were then omitted from downstream analysis. After preprocessing, the final count table retained 14,809 gene entries across 80 samples (20 animals × 4 treatment groups). To ascertain the effect of GR_Ala610Val_ under different treatment conditions, differentially expressed genes (DEGs) in response to the Ala610Val substitution were analyzed separately within each group (CON, DEX, LPS, and LPS+DEX). Two variables—sex (male or female), and GR genotypes (Ala/Ala, Ala/Val, or Val/Val)—were included in the design formula, with the latter as the variable of interest. To calculate the log_2_ fold change (LFC) of genes induced by dosage of the Val allele, the GR genotypes of Ala/Ala, Ala/Val, and Val/Val were expressed as continuous variables with values of zero, one, and two respectively. Genes with a *p*-value <0.05 were considered differentially expressed. Volcano plots were made using the R package EnhancedVolcano version 1.10.0 (22).

### Identification of treatment-specific genes

To further investigate the specific effects of the Val allele under different conditions, the Val-regulated DEGs for the four treatment groups were analyzed using the Venn diagram module in the TBtools toolkit (23). Five gene modules were identified: one module of common Val-regulated DEGs across all four treatment groups (shared genes), and four modules of treatment-specific Val-regulated genes. Furthermore, Val-regulated DEGs unique to the LPS group, and common to the LPS and LPS+DEX groups, were referred to as LPS-specific genes. Similarly, Val-regulated DEGs unique to the DEX group, and common to the DEX and LPS+DEX groups, were referred to as DEX-specific genes. The heatmap was generated using the R package pheatmap version 1.0.12 (24), based on the LFC values of shared genes induced by dosage of the Val allele.

### Identification of Val-up and Val-down genes

Genes exhibiting additive changes in expression associated with the Val allele were identified using the degPatterns function implemented in the R package DEGreport version 1.28.0 (25). To this end, Val-regulated DEGs within each treatment group were subjected to hierarchical clustering. This allowed for identification of genes upregulated (Val-up) or downregulated (Val-down) by the Val allele in an additive manner (i.e., dosage of the Val allele). Within each treatment group, GR targets were identified using the GR targets dataset from Ingenuity Pathway Analysis (IPA; Qiagen, Hilden, Germany) and Harmonizome (26). PCA was conducted based on Val-up and Val-down genes. Separate protein–protein interaction (PPI) networks were constructed for Val-up and Val-down genes, respectively, using the STRING database (27), and visualized by Cytoscape version 3.9.0 (28). Hub genes showing a high degree of connectivity within each PPI network were extracted by the cytoHubba plugin (29).

### Functional enrichment analysis

Functional enrichment analysis was conducted using Metascape (30) and IPA. For Metascape analysis, the ontology sources used were GO Biological Processes, Reactome Gene Sets, KEGG Pathway, and WikiPathways. Metascape terms with a BH-adjusted *p*-value <0.05 were regarded as significantly enriched. When using IPA, z-scores were calculated using the LFC of genes induced by each copy of Val allele within the respective treatment groups. Three types of IPA terms were included in the analysis: canonical pathways, diseases and biological functions, and upstream regulators. IPA terms having a Fisher exact test *p*-value <0.05 and an absolute z-score ≥2 were regarded as having statistical significance and a definite regulation direction. The results of functional enrichment analysis were visualized using GraphPad Prism 8.2.1 (GraphPad Software, Inc., San Diego, CA) and the R package ggplot2 version 3.3.5 (31).

## Results

### Differentially expressed genes associated with the Val allele

**Figure 1:**
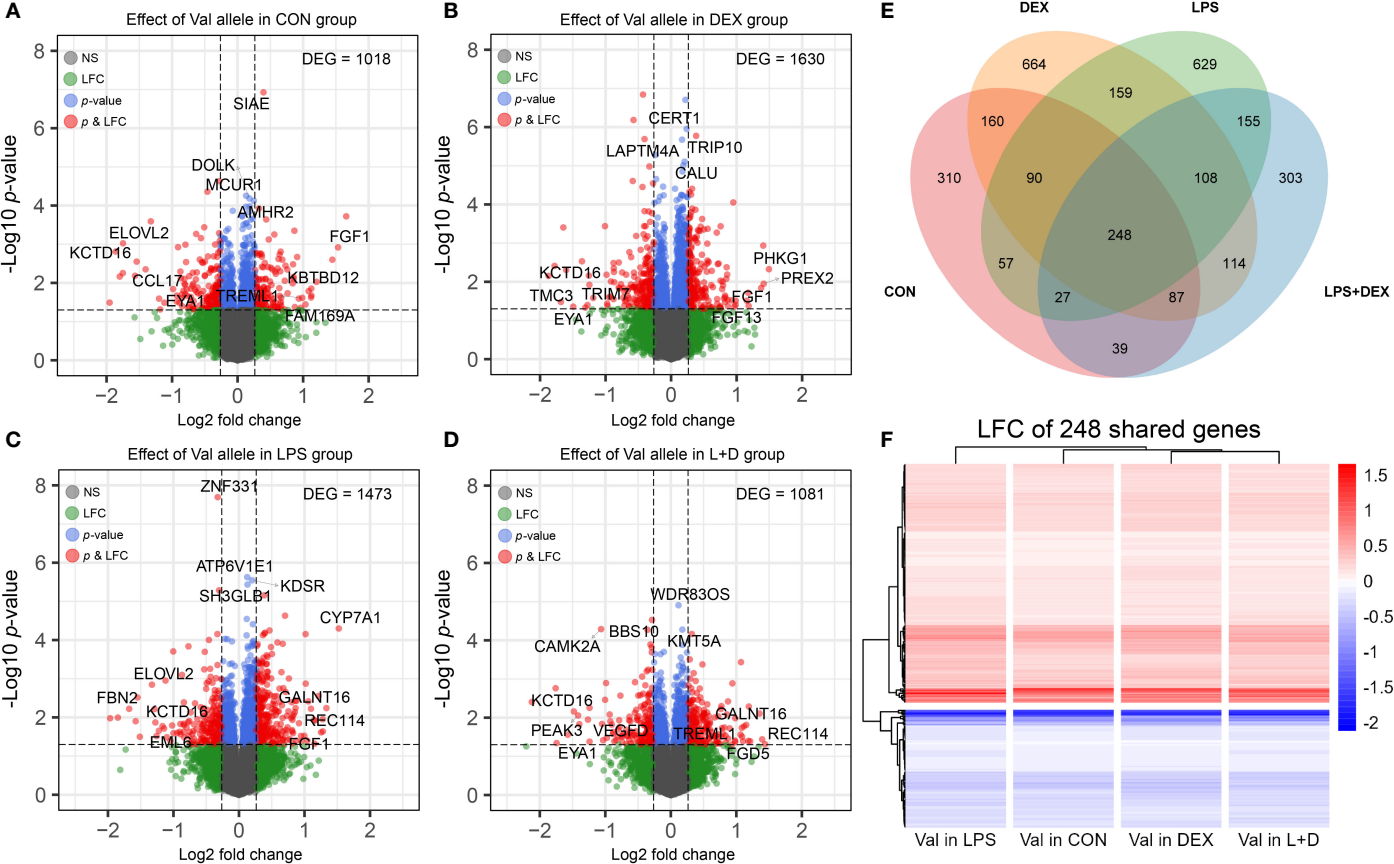
Differential expression analysis. **(A-D)** Volcano plots of differentially expressed genes (DEGs) induced by dosage of the Val allele in **(A)** CON, **(B)** DEX, **(C)** LPS, and **(D)** LPS+DEX groups. The top four most significant (*p*-value) and most up and down regulated DEGs (LFC) were displayed. **(E)** Venn diagram illustrating shared and unique DEGs induced by dosage of the Val allele among four treatment groups. **(F)** Heatmap constructed based on LFC of Val DEGs shared by four treatment groups. Val, Valine; CON, control; DEX: dexamethasone; LPS, lipopolysaccharide; LFC, log2 fold change.

To study the effect of the GR_Ala610Val_ variant on PBMCs in different states of immune activation (CON, DEX, LPS, and LPS+DEX), DEGs associated with per copy of the Val allele were determined within each group. This analysis identified 1018, 1630, 1473, and 1081 DEGs, respectively, in the four treatment groups (**Figures 1A–D**). The top DEGs (according to *p*-values and LFC) are labeled on the volcano plots, and indicate that the Val allele potentially influences immune response (*CAMK2A*, *CCL17*, *SIAE*, *TREML1*, and *TRIM7*); lipid metabolism (*CERT1*, *CYP7A1*, *ELOVL2*, and *KDSR*); protein synthesis and processing (*BBS10*, *CALU*, *DOLK*, *GALNT16*, and *WDR83OS*); the endo-lysosomal system (*ATP6V1E1*, *LAPTM4A*, and *TRIP10*); and cell division and survival (*EYA1*, *FGF1*, *FGF13*, *MCUR1*, *REC114*, *SETD8*, *SH3GLB1*, and *ZNF331*). It is also of note that the top DEG profiles differed between treatment groups, as indicated by the observation of sets of unique DEGs in the CON, DEX, LPS, and LPS+DEX groups (310, 664, 629, and 303, respectively). These data are presented in the Venn diagram (**Figure 1E**; **Table S1**). Nevertheless, there is also a group of shared, constitutive DEGs (248) regulated by the Val allele in the same direction across all four groups (**Figures 1E, F**). These findings demonstrate that the different states of immune activation modulate and manifest the distinct effects of the Val allele on PBMCs, as further emphasized by functional annotation of the DEGs presented below.

### Genes regulated by the Val allele in an additive manner

**Figure 2:**
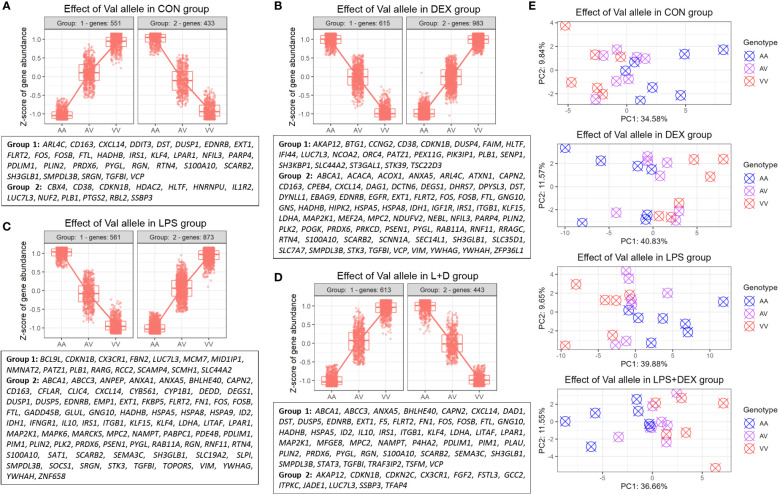
Identification of DEGs regulated by the Val allele additively (Val-up and Val-down genes). **(A-D)** Hierarchical clustering of expression profiles of Val-regulated DEGs in **(A)** CON, **(B)** DEX, **(C)** LPS, and **(D)** LPS+DEX groups. Previously reported glucocorticoid receptor (GR) targets identified through the database of Harmonizome (26) and Ingenuity Pathway Analysis (IPA) were displayed for each cluster. **(E)** Principal component analysis (PCA) of expression profiles of Val-up and Val-down genes in each treatment group. AA, GR genotype AlaAla; AV, GR genotype AlaVal; VV, GR genotype ValVal.

Hierarchical clustering was then applied to the DEGs to identify genes up- or downregulated by the Val allele in an additive manner. The CON, DEX, LPS, and LPS+DEX groups contained 551, 983, 873, and 613 upregulated genes (Val-up), respectively, while 433, 615, 561, and 443 downregulated (Val-down) genes, respectively, were identified (**Figures 2A-D**; **Table S1**). Within each group, PCA revealed that these Val-up and Val-down genes drive the separation in transcriptional programs of the samples bearing different GR genotypes (**Figure 2E**).

Furthermore, many known GR targets were found in these Val-up and Val-down gene clusters (**Figures 2A–D**; **Table S2**). At baseline, without stimulation, Val-up genes included several GR targets involved in inflammatory responses (*CD163*, *CXCL14*, *DUSP1*, *FOS*, *LPAR1*, *NFIL3*, *S100A10*, *SCARB2*, *SMPDL3B*, *SRGN*, and *VCP*), suggesting an intrinsic role of the Val allele in immunomodulation (**Figure 2A**). Additionally, DEX treatment introduced Val-up GR targets related to anti-inflammatory processes (*ANXA5*, *KLF15*, and *RNF11*); lipid metabolism (*ABCA1*, *ACACA*, *ACOX1*, *DEGS1*, *DHRS7*, and *GNS*); mitochondrial function (*IDH1*, *LDHA*, *MPC2*, *NDUFV2*, *PRKCD*, and *VIM*); and protein folding (*CPEB4*, *HSPA5*, and *HSPA8*) (**Figure 2B**). Several distinct Val-up GR targets associated with inflammatory responses (*ANPEP*, *ANXA1*, *DUSP5*, *FKBP5*, *IFNGR1*, *IL10*, *LITAF*, *NAMPT*, *SLPI*, and *SOCS1*) were identified upon LPS stimulation, together with GR targets involved in cell death (*CAPN2*, *CFLAR*, *DEDD*, *EMP1*, *GADD45B*, *GLUL*, *ID2*, *MAP2K1*, *PIM1*, *SAT1*, *STK3*, and *TOPORS*; **Figure 2C**). Considering the role of damage-associated molecular patterns (DAMPs) in coupling cell death to inflammatory responses (32), the identification of these two DEG classes suggested that the Val allele enhances inflammatory signaling associated with the release of DAMPs. Finally, eight Val-up GR targets emerged only when the PBMCs were co-treated with LPS and DEX, namely: *DAD1*, *F5*, *MFGE8*, *P4HA2*, *PLAU*, *STAT3*, *TRAF3IP2*, and *TSFM* (**Figure 2D**).

Notably, many of the Val-down GR targets were related to cell cycle and division, such as *CBX4*, *CDKN1B*, *HDAC2*, *HLTF*, *HNRNPU*, *NUF2*, and *RBL2* (CON group); *BTG1*, *CCNG2*, *DUSP4*, and *ORC4* (DEX group); *MCM7* and *RCC2* (LPS group); and *CDKN2C*, *FGF2*, and *JADE1* (LPS+DEX group; **Figures 2A–D**; **Table S2**). Besides this, DEX stimulation induced additional Val-down GR targets important for function of the GR (e.g. *NCOA2*) and B cells (e.g. *FAIM*, *SH3KBP1*, and *PIK3IP1*; **Figure 2B**).

### Val-up genes reflect enhanced inflammatory signaling associated with LPS stimulation

**Figure 3:**
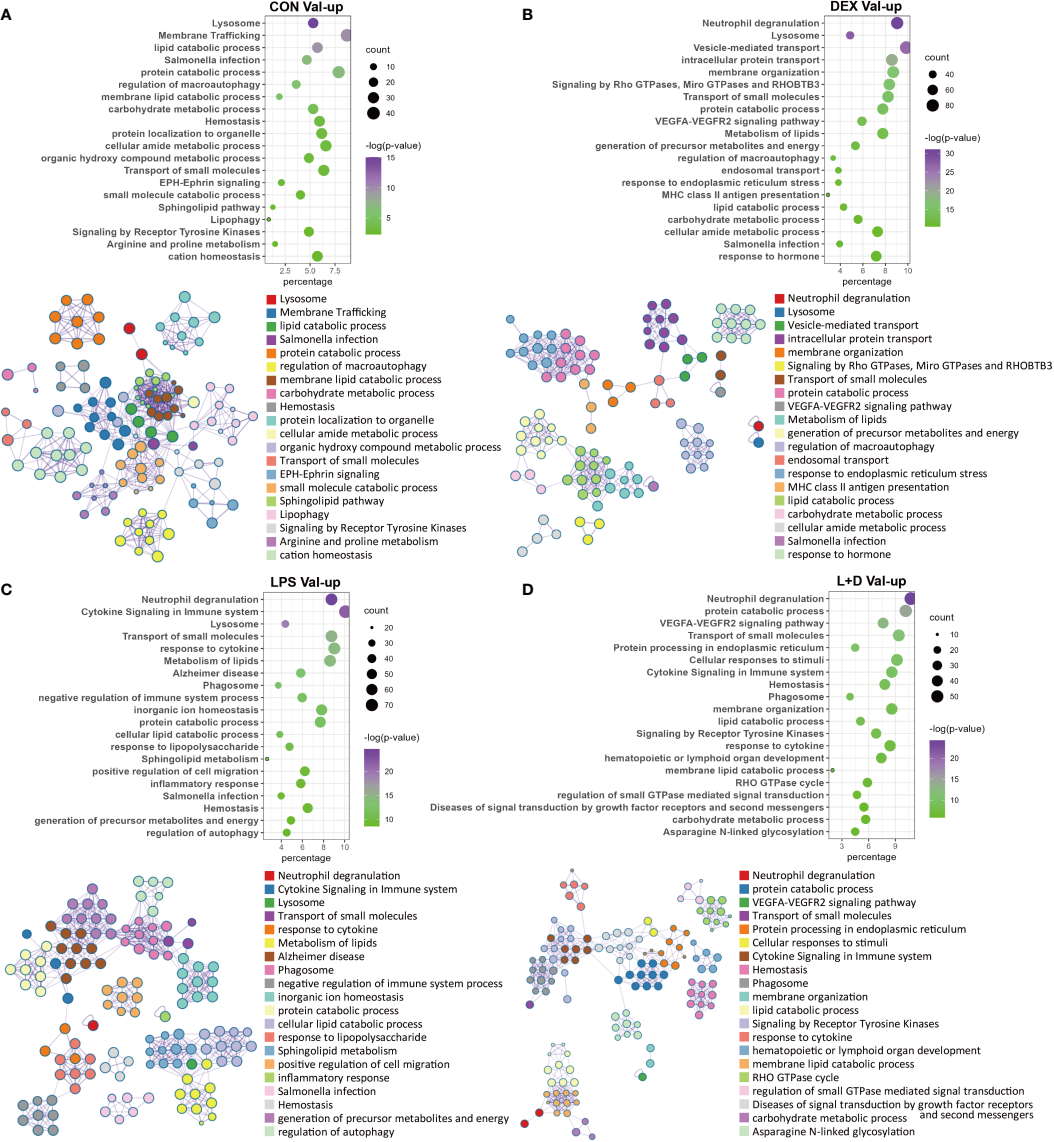
Enrichment analysis of Val-up genes in each treatment group. **(A)** Functional terms enriched for Val-up genes in CON group. **(B)** Functional terms enriched for Val-up genes in DEX group. **(C)** Functional terms enriched for Val-up genes in LPS group. **(D)** Functional terms enriched for Val-up genes in LPS+DEX group. Enrichment analysis was performed using the Metascape. Bubble plots illustrate significance and gene counts of top enriched terms, where purple indicates higher significance and green indicates lower significance and bubble size is positively correlated with gene counts. Networks illustrate the relationship between terms, where each node represents one functional term and terms with Kappa similarities above 0.3 are connected by edges. Each term cluster is labeled and represented by its most significant member and all members in the same term cluster are indicated by the same color.

**Figure 4:**
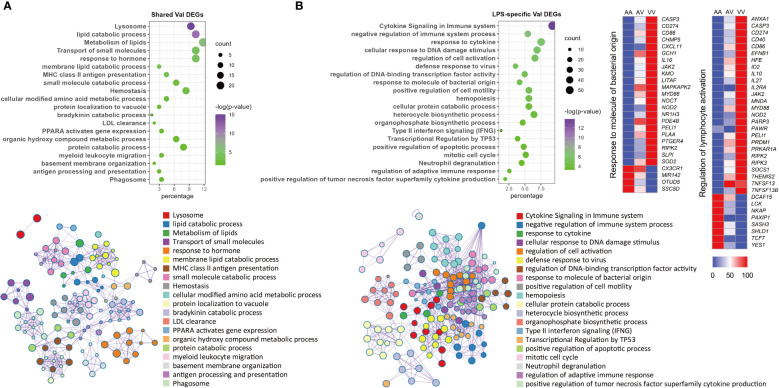
Enrichment analysis of shared and LPS-specific Val-regulated DEGs. **(A)** Functional terms enriched for Val-regulated DEGs common in all the four groups. **(B)** Functional terms enriched for LPS-specific Val-regulated DEGs with relative gene expression profiles for two representative LPS-specific terms. Enrichment analysis was performed using the Metascape. Bubble plots illustrate significance and gene counts of top enriched terms, where purple indicates higher significance and green indicates lower significance and bubble size is positively correlated with gene counts. Networks illustrate the relationship between terms, where each node represents one functional term and terms with Kappa similarities above 0.3 are connected by edges. Each term cluster is labeled and represented by its most significant member and all members in the same term cluster are indicated by the same color.

Because of their distinct implications, enrichment analysis was carried out independently for Val-up and Val-down genes. The Val-up genes exhibited a variety of functions with many of them common to non-stimulated and stimulated conditions, and thus taken to represent constitutive roles of the Val allele. For example, in common DEGs these functional terms included lysosome, protein and lipid catabolic processes, transport of molecules, and ion homeostasis. They also encompassed a series of signaling pathways involved in immune responses: neutrophil degranulation, MHC mediated antigen processing and presentation, platelet activation, sphingolipid metabolism, and *Salmonella* infection (**Figures 3A–D**; **Table S3**). Accordingly, when analyzing treatment-specific genes, Val DEGs common to all treatment groups supported the above constitutive roles of the Val allele (**Figure 4A**; **Table S4**). Consistent with this, the IPA disease and function database predicted that lysosomal storage disease and accumulation of sphingolipid were inhibited, whereas inflammatory response and endocytosis were activated for Val-up genes in all four groups (**Table S5**).

Two additional types of pathway emerged for Val-up genes upon treatment with DEX. Specifically, these were related to protein processing in the endoplasmic reticulum (ER) and mitochondrial energy generation (**Figure 3B**; **Table S3**). Additional support was again provided by IPA predictions: activation of the unfolded protein response, TCA cycle, mitochondrial dysfunction, and production of reactive oxygen species (ROS; **Tables S5**, **S6**). These results indicate a potential way of the Val allele in regulating cellular activities, since mitochondria have been shown to contact lysosomes and the ER, and these interactions are essential for many cellular functions including cell fate decisions (33).

Compared with DEX, the Val allele exhibited genuine, albeit weaker, influence on mitochondrial dysfunction and unfolded protein response when stimulated with LPS (**Table S6**). Notably, LPS also triggered a set of immune pathways found among the Val-up genes, including cytokine signaling, response to LPS, T cell receptor (TCR) signaling, B cell receptor (BCR) signaling, and ROS and RNS production in phagocytes (**Figure 3C**; **Table S3**). Accordingly, aside from the activated terms already identified for the CON and DEX groups—such as antigen presentation, endocytosis, phagocytosis, and leukocyte migration—the Val-up genes of the LPS group included an additional series of predicted terms involving activation of lymphocytes and T cells (**Table S5**). Furthermore, IPA predictions identified multiple molecules crucial for inflammatory signaling as activated upstream regulators for Val-up genes in the presence of LPS (such as CD40, EIF2AK2, IL27, JAK2, MYD88, NOD2, RIPK2, STAT2, TLR4, and TNFSF13B), with their predicted activation also corresponding to increased expression under control of the Val allele (**Table S1**; **Table S7**). The above results suggest that the Val allele might amplify or skew cellular responses to pro-inflammatory factors like LPS, resulting in a more severe inflammatory phenotype associated with enhanced PRR-mediated inflammatory signaling and signal transduction from antigen-presenting cells (APCs) to T cells. This was apparently confirmed by the identification of a suite of immune mediators, along with enrichment of various immune signaling pathways, among the LPS-specific Val-mediated DEGs. These included response to molecules of bacterial origin, and regulation of lymphocyte activation (**Figure 4B**; **Table S4**).

Another key finding for Val-up genes in the LPS group was enrichment of programmed cell death (**Table S3**), which was further categorized by the IPA database into different subtypes, revealing that ferroptosis, autophagy, and necroptosis were activated by the Val allele, whereas apoptosis was inhibited (**Table S5**; **Table S6**). This might be associated with the enriched terms of iron homeostasis, ER stress, mitochondrial dysfunction, and fatty acid oxidation (34) (**Table S6**). In fact, some of these cell death terms were already identified in the CON and DEX groups (**Table S6**). However, cell death signaling induced by LPS might have more severe consequences due to increased expression of PRRs such as *DDX58* and *NOD2* (**Table S1**), because DAMPs released by damaged cells are tightly associated with inflammation *via* their recognition by PRRs (32).

Several enriched terms in the LPS+DEX group rationalized the improved response to DEX therapy seen in Val carriers during endotoxemia (16). For example, activation of necroptosis was identified among the Val-up genes in both the LPS and DEX groups, but was not enriched in the LPS+DEX group (**Table S6**). In contrast, necrosis was predicted by IPA to be inhibited exclusively in this latter group (**Table S5**). The VEGFA–VEGFR2 signaling pathway plays a key role in the activation of endothelial nitric oxide synthase (eNOS) and eNOS-mediated nitric oxide (NO) production (35), which has been found to be significantly reduced in sepsis (36). This pathway was enriched only in the presence of DEX (DEX and LPS+DEX groups; **Figures 3B, D**). Several molecules with pivotal roles in anti-inflammatory responses—such as IL6R (37), IRF2 (38), KLF4 (39), and NCOA3 (40)—were predicted as activated upstream regulators only when co-treated with LPS and DEX (**Table S7**).

### Val-down genes represent cell cycle and division processes independent of treatment

**Figure 5:**
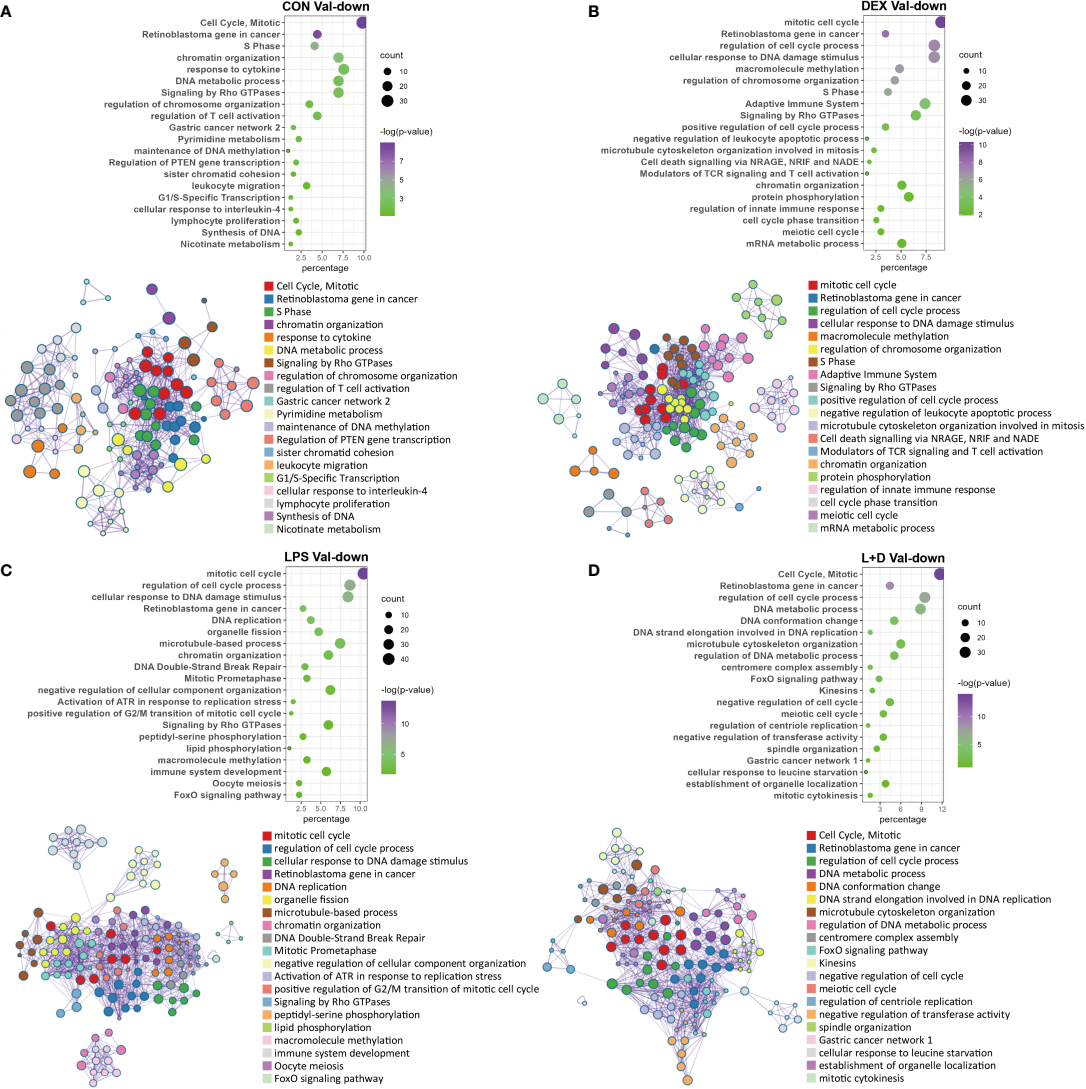
Enrichment analysis of Val-down genes in each treatment group. **(A)** Functional terms enriched for Val-down genes in CON group. **(B)** Functional terms enriched for Val-down genes in DEX group. **(C)** Functional terms enriched for Val-down genes in LPS group. **(D)** Functional terms enriched for Val-down genes in LPS+DEX group. Enrichment analysis was performed using the Metascape. Bubble plots illustrate significance and gene counts of top enriched terms, where purple indicates higher significance and green indicates lower significance and bubble size is positively correlated with gene counts. Networks illustrate the relationship between terms, where each node represents one functional term and terms with Kappa similarities above 0.3 are connected by edges. Each term cluster is labeled and represented by its most significant member and all members in the same term cluster are indicated by the same color.

In contrast to the myriad functions exhibited by Val-up genes, these were less diverse for the Val-down genes. The most obvious feature was their consistent association with inhibition of cell cycle progression and cell division, regardless of the treatment group (**Figures 5A–D**). This classification harbored a variety of pathways, involving cell cycle regulation and phase transition, chromatin and cytoskeleton organization, DNA replication and repair, and organelle fission (**Figures 5A–D**). These effects on cell cycle and cell division were further confirmed by IPA, which predicted inhibition of cell cycle control of chromosomal replication, organization of cytoskeleton, repair of DNA, transcription, and segregation of chromosomes (**Table S5**; **Table S6**). Besides this, several positive regulators of cell cycle progression, such as CKAP2L (41), E2F1 (42), and EP400 (43), were predicted as inhibited upstream regulators (**Table S7**), whereas activation was predicted for the retinoblastoma (Rb) protein, which plays a pivotal role in negative regulation of the cell cycle (44).

Another key theme that emerged among Val-down genes was negative regulation of the immune system. In the absence of LPS (CON and DEX groups), Val-down genes were found to be related to both innate and adaptive immune signaling, with the former mainly involving response to cytokines and the latter associated with T cells (**Figures 5A, B**). These results were supported by IPA enrichment, which showed inhibition of a series of terms implicated in the quantity, homeostasis, maturation, and development of T cells (**Table S5**). In addition, inflammatory mediators such as LPS and IFNG were predicted by IPA as inhibited upstream regulators in the CON and DEX groups, while Irgm1—which has been found to prevent endotoxemia in mice (45)—was predicted to be activated under these conditions (**Table S7**). There is currently no report describing Irgm1 in the pig, however, its predicted activation indicates that Val-down genes altogether induce anti-inflammatory signaling.

In LPS treatment, none of the above inflammatory mediators (LPS, IFNG, and Irgm1) were enriched for the Val-down genes (**Table S7**), indicating that LPS abolished Val allele-mediated anti-inflammatory responses. However, activation of the anti-inflammatory factor Irgm1 was predicted under co-treatment with LPS and DEX (**Table S7**).

### Hub genes support the key functions of Val-up and Val-down clusters

**Figure 6:**
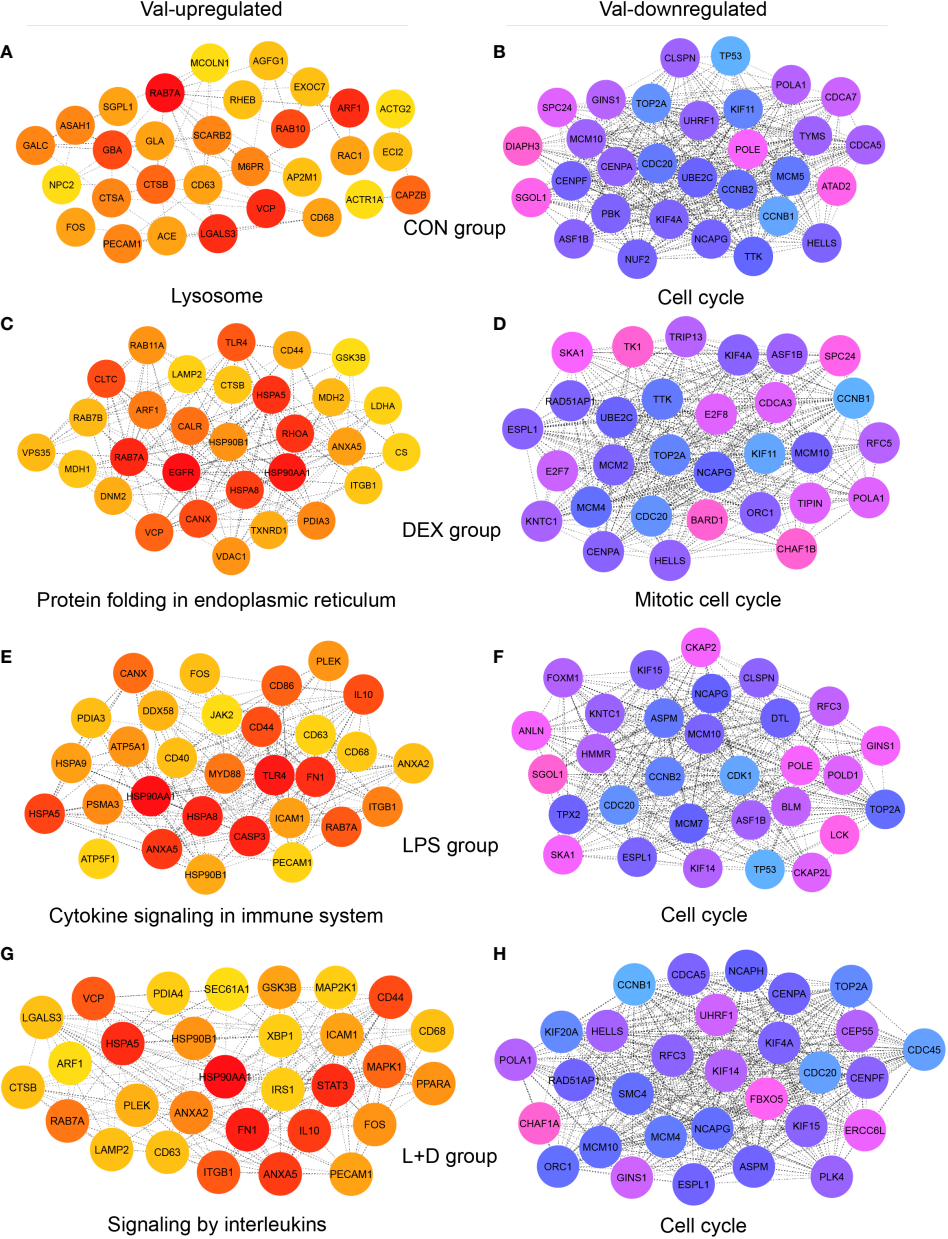
Identification of hub genes of Val-up and Val-down clusters in each treatment group. **(A-H)** Protein-protein interaction (PPI) networks showing top 30 hub genes with high connectivity within **(A)** Val-up cluster in CON group, **(B)** Val-down cluster in CON group, **(C)** Val-up cluster in DEX group, **(D)** Val-down cluster in DEX group, **(E)** Val-up cluster in LPS group, **(F)** Val-down cluster in LPS group, **(G)** Val-up cluster in LPS+DEX group, and **(H)** Val-down cluster in LPS+DEX group. PPI networks were constructed by the STRING database (27) and top 30 hub genes of each network were identified by the Cytoscape plugin cytoHubba (28, 29). Connectivity was correlated with color of circles where red and blue indicate higher degree whereas yellow and pink indicate lower degree. The most significant functional terms of hub genes of each cluster were identified using the Metascape (30) and displayed.

Hub genes, meaning genes showing a high degree of connectivity in PPI networks of Val-regulated DEGs, also exhibited different profiles among treatment groups and supported the key functions of respective clusters (**Figure 6**; **Table S2**). Without stimulation, the identified Val-up hub genes were associated with lysosomal function (*CD68*, *CTSA*, *CTSB*, *M6PR*, *MCOLN1*, *NPC2*, and *SCARB2*); vesicle-mediated transport (*ACTR1A*, *AGFG1*, *AP2M1*, *ARF1*, *CD63*, *EXOC7*, *RAB10*, *RAB7A*, and *RAC1*); and sphingolipid metabolism (*ASAH1*, *GALC*, *GBA*, *GLA*, and *SGPL1*; **Figure 6A**). In comparison, many Val-up hub genes in the DEX group were functionally distinct, and were related to protein folding, processing, and transport (*CALR*, *CANX*, *CLTC*, *DNM2*, *HSP90B1*, *HSPA5*, *HSPA8*, *LAMP2*, *PDIA3*, *RAB11A*, *RAB7B*, and *VPS35*) and mitochondrial respiration (*CS*, *GSK3B*, *LDHA*, *MDH1*, *MDH2*, and *VDAC1*; **Figure 6C**). Stimulation by LPS induced hub genes related to pattern recognition receptor (PRR) and inflammatory signaling (*ANXA2*, *ANXA5*, *CD40*, *CD44*, *CD86*, *DDX58*, *FN1*, *HSP90AA1*, *ICAM1*, *IL10*, *JAK2*, *MYD88*, and *TLR4*; **Figure 6E**). Given the cell death-related GR targets and signaling pathways found in this cluster, and the role of PRRs in sensing DAMPs as well as PAMPs (46), the hub genes found here apparently confirm Val allele-induced amplification of inflammatory signaling resulting from DAMP and PAMP recognition by PRRs. In the LPS+DEX group, two anti-inflammatory hub genes were identified—*STAT3* (47) and *PPARA* (48) (**Figure 6G**)—which might partially explain the superior response to DEX therapy in Val carriers among endotoxin-challenged pigs (16).

Most of the 30 Val-down hub genes identified in the four treatment groups centered unanimously around cell cycle, mitosis, and DNA replication, suggesting substantial inhibition of these processes (**Figures 6B, D, F, H**). Among them, *CDC20*, *MCM10*, *NCAPG*, and *TOP2A* were shared by all groups. Each group also contained unique hub genes, including *ATAD2*, *CDCA7*, *DIAPH3*, *MCM5*, *NUF2*, *PBK*, and *TYMS* (CON group); *BARD1*, *CDCA3*, *CHAF1B*, *E2F7*, *E2F8*, *MCM2*, *RFC5*, *TIPIN*, *TK1*, and *TRIP13* (DEX group); *ANLN*, *BLM*, *CDK1*, *CKAP2*, *CKAP2L*, *DTL*, *FOXM1*, *HMMR*, *MCM7*, *POLD1*, and *TPX2* (LPS group); and *CDC45*, *CEP55*, *CHAF1A*, *ERCC6L*, *FBXO5*, *KIF20A*, *NCAPH*, *PLK4*, and *SMC4* (LPS+DEX group; **Figures 6B, D, F, H**).

## Discussion

This study has focused on determining the impact of a previously discovered natural Ala610Val substitution in the porcine glucocorticoid receptor (GR_Ala610Val_) (49) on the transcriptional profiles of porcine PBMCs under non-stimulated and stimulated conditions. By utilizing PBMCs carrying all three GR genotypes (Ala/Ala, Ala/Val, and Val/Val), we identified a subset of genes whose transcription is altered by the Val variant in an additive manner, thus showing a genuine genetic effect. The resultant findings provide a clear evidence for a distinct transcriptome signature of the Val allele, which alters the response to immune stimuli, as seen through upregulated inflammatory responses and downregulation of cell cycle progression and cell division. These results offer a possible explanation and mutual support for the aggravated consequences of LPS-induced endotoxemia associated with the GR_Ala610Val_ variant (16), as in more detail elaborated below.

Among the various functions being represented by the Val-up genes, enhanced inflammatory response stands out as the most prominent theme. This is evidenced by the enrichment of signaling pathways involving endocytosis, antigen presentation, and particularly, PRR-mediated recognition and signal transduction of DAMPs and PAMPs among Val-up DEGs.

The first critical impact of the Val allele on immune signaling is upregulation of genes involved in endocytosis and antigen presentation. The three major types of endocytosis are pinocytosis, receptor-mediated endocytosis, and phagocytosis, of which the latter serves as a front-line defense against microbes, and is a crucial function of professional phagocytes including macrophages, monocytes, and neutrophils (50). In this process, extracellular microbial materials are internalized into phagosomes and ultimately subjected to degradation in the lysosome. Endocytosis and lysosomal degradation are both required for effective pathogen elimination (51). The Rab GTPases, such as Rab5 and Rab7, play key regulatory roles in endocytosis and exhibit substantial interactions with immune signals (50). Rab5 regulates fusion of phagosomes with early endosomes, whereas Rab7 facilitates the fusion of late endosomes and phagosomes with lysosomes; and both proteins can be upregulated in response to cytokine stimulation (50). In this study, we found that *RAB5C*, *RAB7A*, and *RAB7B* were all upregulated by the Val allele, supporting increased constitutive microbe internalization induced by the GR variant. Along with endocytosis, lysosomal functioning was also significantly enriched among Val-up genes. This is consistent with the Val-associated upregulation of *ATP6V1E1*, which encodes a subunit of the vacuolar ATPase (V-ATPase), an ATP hydrolysis-driven proton pump crucial for acidification of lysosomes (52).

Aside from endocytosis, the lysosome is also associated with antigen presentation. Professional APCs such as dendritic cells (DCs), B cells, and macrophages all constitutively express major histocompatibility complex (MHC) class II molecules (53). They internalize and process antigens, then present antigenic peptides to T cells (54). The antigen processing step relies on proteolysis in endosomal–lysosomal antigen-processing compartments, which is tightly regulated in APCs to generate proper peptides (53). In the current study, we found that MHC-mediated antigen presentation was enriched for Val-up genes regardless of treatment, and corresponded with enrichment of lysosomal terms. Antigen presentation is usually considered important for initiating T cell-dependent adaptive responses, but many studies have also observed interplay with TLR-mediated innate immune signaling (55). For example, LPS treatment increased MHC class II expression in DCs, in a manner dependent on an AP-1 enhancer (56), whereas deficiency in MHC class II molecules alleviated TLR-induced cytokine production and endotoxic shock (57). Further investigation revealed that intracellular MHC class II molecules interact with the tyrosine kinase Btk and promote its activation, which facilitates TLR signaling *via* interaction with MyD88 and TRIF (57). Furthermore, Btk activation requires a costimulatory molecule, CD40 (57), which was identified as a Val-up gene in our study, but only in the presence of LPS.

The major finding of this study is the upregulation of LPS signaling pathway, including TLR4 and its related molecules, by the Val variant. Toll-like receptors (TLRs) are one of the most widely studied molecules in the innate immune system. In this study, we found that the Val allele upregulated *TLR4*, *TLR8*, and *CD180* of the TLR family. TLR4 is the main receptor for LPS (58). TLR4 signaling in response to LPS relies on cooperation of myeloid differentiation factor 2 (MD2) and CD14, subsequently leading to stimulation of MyD88-dependent and TRIF-dependent signaling, in turn causing activation of NF-κB, AP-1, MAPK, and IRF3 cascades (59). Along with *TLR4*, other key molecules in TLR4-LPS signaling such as *MYD88* and *LY96* (encoding MD2) were identified as Val-up genes. Among these, *LY96* was constitutively upregulated by the Val allele across treatments, perfectly in line with the predisposition of Val carriers to enhanced LPS response. Unlike *LY96*, *MYD88* was affected by Val only in the presence of LPS. *CD180* mediates B cell activation by LPS (60). *CD180* enhances TLR4 signaling in B cells but inhibits it in macrophages and DCs; the opposite effect appears associated with TLR4 abundance (61). Thus, *CD180* likely contributes to Val enhancement of LPS signaling in porcine B cells. LPS signaling molecules upregulated by the Val allele include *TIRAP*, *FOS*, *HSP90AA1*, *LITAF*, *PELI1*, and *PLAA*. *HSP90AA1* was identified as a Val-up GR target gene in our study. Among other functions, HSP 90-alpha was implicated in LPS signal transduction by serving as a CD14-independent LPS receptor (62). *PELI1* encoding an E3 ubiquitin ligase participates in LPS-mediated induction of CD86 and MHCII in B cells and Its deficiency renders mice resistant to LPS-induced septic shock (63). Thus, upregulation of TLR4 and its associated molecules by the Val variant is proposed here as the central mechanism for the enhanced response of Val carriers to endotoxemia observed in the *in vivo* LPS challenge previously (16).

In addition to TLR4 signaling, the Val allele upregulated many other genes and pathways implicated in PRR signaling, particularly in the LPS group, such as *DDX58* and *NOD2*. The first of these, *DDX58*, encodes retinoic acid-inducible gene-I (RIG-I), an intracellular PRR responsible for sensing viral nucleic acids (58). When activated, RIG-I binds mitochondrial antiviral signaling protein (MAVS), a key adaptor protein that can mediate downstream activation of the NF-κB and IRF3 signaling pathways (64). Nucleotide binding oligomerization domain containing 2 (NOD2) is another intracellular PRR that recognizes muramyl dipeptide (MDP) in the cell wall of both Gram-negative and Gram-positive bacteria (58). Activation of NOD2 also triggers NF-κB signaling, through recruitment of the downstream receptor-interacting protein kinase 2 (RIPK2) (65); however, NOD2 signaling requires the decomposition of cell walls by lysosomes (58). This apparently confirms the contribution of Val-mediated upregulation of lysosomal functions to enhanced immune signaling. Furthermore, upregulation of *DDX58*, *NOD2*, and *RIPK2* by the Val allele was found only in the presence of LPS, indicating that LPS serves as a trigger to amplify Val upregulation of inflammation.

Upregulation of the above PRRs may also contribute to enhanced inflammatory signaling *via* DAMPs released by damaged cells, since the Val allele activated autophagy, ferroptosis, and necroptosis. Autophagy is a parallel process to the lysosomal degradation of cellular materials (66), and can also be induced by ER stress (67). Increased autophagy is therefore in agreement with the enrichment of lysosome, ER stress, and unfolded protein response pathways that were identified among Val-up genes. Ferroptosis is an iron-dependent cell death pathway accompanied by lipid peroxidation and ROS generation (34), and its upregulation fits with the observed enrichment of fatty acid β-oxidation, mitochondrial dysfunction, and iron homeostasis signaling pathways. Necroptosis is a regulated inflammatory mode of cell death, which critically relies on the participation of RIPK3 and mixed lineage kinase domain-like protein (MLKL). To initiate necroptosis, RIPK3 recruits and activates MLKL by phosphorylating its pseudokinase domain, which promotes oligomerization. Following this, oligomerized MLKL translocates to the plasma membrane, where its pore-forming properties cause membrane damage (34). In our study, *MLKL* was identified as a Val-up gene independent of treatment, whereas *RIPK3* was only observed in the LPS group. Therefore, as seen for inflammatory processes, LPS serves as an external signal to promote cell death. The aforementioned Val-up GR target gene *HSP90AA1* also facilitates necroptosis, since it encodes a chaperone protein required for oligomerization and translocation of MLKL (68).

Cell death signaling interacts substantially with PRRs. DAMPs released by damaged cells can activate PRRs, thereby triggering inflammatory responses involving migration and phagocytosis of immune cells, and production of cytokines (46). These DAMPs can be derived from various cellular compartments—such as mitochondria, the cytosol, and nucleus—and include a range of molecular classes such as HMGB1, IL-1α, uric acid, S100 proteins, heat shock proteins, ATP, mROS, RNA, DNA, and nucleotides (32). According to this, we found that the Val allele upregulated many genes encoding heat shock proteins and S100 proteins, such as *HSPA5*, *HSPA8*, *HSPA9*, *HSP90AA1*, *S100A10*, *S100A6*, and *S100Z*. The DAMPs can be recognized by different PRRs, including those upregulated by the Val allele (NOD2, RIG-I, and TLRs), to initiate specific inflammatory signaling (67). In turn, the activation of different types of PRRs can induce cell death (46). For example, activation of TLR4 by LPS stimulates necroptosis in mouse macrophages, in a process involving assembly of a TRIF–RIPK3 complex and accumulation of ROS (69).

Val-up genes also indicated activation of several specific pathways involved in cell types not included in this study, which may be regulated by the Val allele in shared pathways also in those cell types. Notably, among these are also pathways related to platelet function. Apart from their key functions in hemostasis, platelets play an essential role in inflammatory diseases and development of sepsis (70). They exert their immunomodulatory functions through interaction with immune cells including neutrophils, or production of immune mediators like cytokines (71). Persistent reduction in platelet numbers is an established risk factor for mortality in sepsis (70). The Val allele-dependent upregulation of several genes associated with platelet activation supports our previous observation that platelet numbers drop more significantly in GR_Ala610Val_ pigs during endotoxemia (16).

Interestingly, our *in vivo* study revealed that DEX rescue of LPS-induced platelet reduction was more effective in Val carriers (16). This might be partially explained by Val-mediated upregulation of the VEGFA–VEGFR2 signaling pathway, which was enriched only in the presence of DEX (i.e., DEX and LPS+DEX groups). This pathway is important in various functions of endothelial cells, such as survival, proliferation, migration, and particularly, eNOS-mediated NO production (35). Specifically, phosphorylation of VEGFR-2 is essential for VEGF-stimulated NO release from endothelial cells, due to its implication in Akt-dependent eNOS activation (72). VEGFR-2 also facilitates VEGF-stimulated eNOS association with the chaperone protein Hsp90 (73), and it has been reported that overexpression of eNOS in endothelial cells inhibits platelet aggregation (74) through NO stimulation of soluble guanylate cyclase in platelets. This increases intracellular levels of cGMP, which represses platelet aggregation and adhesion (75). The role of NO in sepsis is complex. Whereas it is toxic to microbes and helps reduce tissue injury, excessive NO generation promotes sepsis pathogenesis (76). Deficient endothelial NO production is also detrimental, and is believed to be a driver of microvascular dysfunction in septic shock (76). This is supported by observations that inducible nitric oxide synthase (iNOS)-derived NO production is elevated in sepsis, whereas the amount formed by eNOS is reduced, resulting in overwhelming platelet aggregation and leukocyte adhesion (36).

Another significant facet of alteration of porcine PBMCs in Val carriers is inhibition of cell cycle progression, as represented by Val-down genes. In this study, IPA predicted that E2F1 was inhibited whereas Rb was activated. E2F1 is a TF controlling expression of many key genes implicated in G1/S transition. It is negatively regulated by Rb *via* heterodimerization (42), although inhibition of E2F1 is known to cause developmental anomalies and dampen DNA repair (77). Phosphorylation of Rb by cyclin-dependent kinases suppresses its association with E2F1, leading to recovery of TF activity (42). In addition to diminishing E2F1 function, Rb represses the expression of cell cycle genes *via* interacting with proteins needed for nucleosome remodeling and histone modification. In doing so, it induces changes in chromatin structure (44). Several studies have revealed that cell cycle arrest is associated with ferroptosis (78), necroptosis (79), and autophagy (80). Further investigation is therefore warranted, to establish if and how Val allele-mediated inhibition of cell cycle progression contributes to enhanced cell death and inflammatory responses.

Despite the enrichment of immune terms discussed above, our previous work has shown that in unstimulated condition, i.e. in untreated pigs, most inflammatory parameters are not significantly affected by GR_Ala610Val_ *in vivo* (16). This might be related to a subset of Val-affected genes involved in negative regulation of immune responses. Without LPS stimulation, response to cytokines was enriched for Val-down genes, which included the pro-inflammatory genes *CCL17*, *CD38*, *IL18RAP*, *IRF1*, *IRF7*, *ITGA4*, *PTGS2*, and *XCL1*. None of these genes was regulated by the Val allele in the LPS group. In the CON and DEX groups, LPS and IFNG were predicted by IPA as both activated (for Val-up genes) and inhibited (for Val-down genes) upstream regulators, whereas in the LPS group, only their activation was observed. In the absence of LPS, a possible alternative mechanism to counterbalance enhanced immune signaling is inhibition of T cell activation. This was seen among the Val-down genes in IPA-predicted inhibition of T cell-associated terms. Professional APCs recognize and bind targets *via* PRRs then transfer signals to T cells, thus providing a bridge between innate and adaptive immunity (81). When treated with LPS, the Val-up genes featured multiple activated T cell pathways in contrast to baseline conditions. These results indicate that without LPS, the Val allele mediates both activation and inhibition of immune signaling to maintain homeostasis. However, this homeostasis is disrupted in the presence of LPS stimulus.

## Conclusion

The main goal of this study was to elucidate mechanisms behind the enhanced LPS sensitivity of Val carriers observed previously in our *in vivo* challenge. The discovered transcriptional signature suggests that the Val allele affects various immune-related functions in porcine PBMCs. Among the different functional alterations caused by the Val variant stand out its effects on inflammatory signaling associated with PRRs, above all TLR4, and cell death linked with DAMPs, as the determining mechanisms for an enhanced LPS sensitivity and response. In addition, upregulation of the different PRRs, such as *DDX58* and *NOD2*, together with the effect on several other immune functions, suggests that GR_Ala610Val_ may have a broader impact on disease susceptibility in pigs. Overall, the present results emphasize the value of the GR_Ala610Val_ pigs as a unique animal model to explore fundamental biology of GR signaling on the one hand, and for the research to improve pig health on the other.

## Data availability statement

The datasets presented in this study can be found in online repositories. The names of the repository/repositories and accession number(s) can be found below: https://www.ebi.ac.uk/arrayexpress/, E-MTAB-9808.

## Author contributions

EM: Conceptualization and Funding acquisition. EM and ZL: Methodology. ZL: Investigation and Formal analysis. FH: Data Curation. FH: Software. KW: Supervision. ZL and EM: Writing - original draft. All authors contributed to manuscript revision, read, and approved the submitted version.

## Funding

The current study was funded by a grant from the German Research Foundation (Deutsche Forschungsgemeinschaft (DFG)—Project number 391382814), and matched funding from the FBN.

## Acknowledgments

The authors thank Angelika Deike, Marlies Fuchs, Angela Garve, and Janine Wetzel for excellent technical help and the whole staff of the experimental farm (EAS) for animal management and care.

## Conflict of interest

The authors declare that the research was conducted in the absence of any commercial or financial relationships that could be construed as a potential conflict of interest.

## Publisher’s note

All claims expressed in this article are solely those of the authors and do not necessarily represent those of their affiliated organizations, or those of the publisher, the editors and the reviewers. Any product that may be evaluated in this article, or claim that may be made by its manufacturer, is not guaranteed or endorsed by the publisher.
